# Permanent stoma rates after anterior resection for rectal cancer: risk prediction scoring using preoperative variables

**DOI:** 10.1093/bjs/znab260

**Published:** 2021-09-11

**Authors:** E Back, J Häggström, K Holmgren, M M Haapamäki, P Matthiessen, J Rutegård, M Rutegård

**Affiliations:** 1 Department of Surgical and Perioperative Sciences, Surgery, Umeå University, Umeå, Sweden; 2 Department of Statistics, Umeå School of Business, Economics and Statistics, Umeå University, Umeå, Sweden; 3 Department of Surgery, Faculty of Medicine and Health, School of Health and Medical Sciences, Örebro University, Örebro, Sweden; 4 Wallenberg Centre for Molecular Medicine, Umeå University, Umeå, Sweden

## Abstract

**Background:**

A permanent stoma after anterior resection for rectal cancer is common. Preoperative counselling could be improved by providing individualized accurate prediction modelling.

**Methods:**

Patients who underwent anterior resection between 2007 and 2015 were identified from the Swedish Colorectal Cancer Registry. National Patient Registry data were added to determine presence of a stoma 2 years after surgery. A training set based on the years 2007–2013 was employed in an ensemble of prediction models. Judged by the area under the receiving operating characteristic curve (AUROC), data from the years 2014–2015 were used to evaluate the predictive ability of all models. The best performing model was subsequently implemented in typical clinical scenarios and in an online calculator to predict the permanent stoma risk.

**Results:**

Patients in the training set (*n* = 3512) and the test set (*n* = 1136) had similar permanent stoma rates (13.6 and 15.2 per cent). The logistic regression model with a forward/backward procedure was the most parsimonious among several similarly performing models (AUROC 0.67, 95 per cent c.i. 0.63 to 0.72). Key predictors included co-morbidity, local tumour category, presence of metastasis, neoadjuvant therapy, defunctioning stoma use, tumour height, and hospital volume; the interaction between age and metastasis was also predictive.

**Conclusion:**

Using routinely available preoperative data, the stoma outcome at 2 years after anterior resection for rectal cancer can be predicted fairly accurately.

## Teaser

A permanent stoma is common after anterior resection for rectal cancer. Using national Swedish registry data, a prediction model was derived and validated, with moderate ability to predict the stoma fate. Online calculators incorporating this model might improve preoperative counselling.

## Introduction

Anterior resection with restoration of gastrointestinal continuity is the standard of care in the surgical treatment of rectal cancer, provided that it is oncologically safe and the patient does not have severe medical or functional co-morbidity that precludes anastomosis. Despite the desired aim to restore gastrointestinal continuity, long-term permanent stoma rates are reported in 18–25 per cent of patients^1–6^. Anastomotic leakage is a major risk factor for a permanent stoma^1–3^. Proximal defunctioning stomas are commonly used to protect against the consequences of a leak^7^^,^^8^. Nevertheless, around 20 per cent of defunctioning stomas are never closed or are converted to an end colostomy^1^^,^^9–11^, possibly contributing to higher than desired permanent stoma rates.

The possibility that a patient’s treatment may culminate in a permanent stoma, even if not initially planned, should be discussed ahead of rectal cancer surgery. Most previous research in this area has focused on factors that predict non-reversal of defunctioning stomas^1^^,^^2^^,^^9^^,^^12^^,^^13^. The aim of this population-based study was to construct a permanent stoma risk prediction model, using risk factors known before surgery for all patients in whom anterior resection for rectal cancer was planned, regardless of planned use of a defunctioning stoma.

## Methods

This study was approved by the ethical review board at Umeå University, Umeå, Sweden. Patients who had elective anterior resection for rectal cancer in Sweden between 1 January 2007 and 31 December 2015 were identified from the Swedish Colorectal Cancer Registry (SCRCR). Exclusion criteria were: death before 2-year follow-up, treatment at hospitals that no longer performed rectal cancer surgery, and a registered tumour height below 3 cm from the anal verge; the latter criterion was applied because such low tumour heights might be due to a registration error regarding this variable or procedure, as sphincter-saving surgery would be unlikely in this scenario. The SCRCR has been validated multiple times, demonstrating near-complete coverage^14–16^. It is continually cross-checked against the National Cancer Registry for missing registrations. Reporting to the registry includes patient demographics, surgical details, postoperative course, final pathological assessment, and also 5-year follow-up. Rectal cancer is defined by the registry as a large bowel adenocarcinoma, located at least partially within 15 cm from the anal verge, measured using rigid sigmoidoscopy.

### Registry-based stoma outcome

Patients were followed until 31 December 2017 regarding stoma outcome. The SCRCR was used to determine whether patients had received a defunctioning stoma. In brief, procedure codes for all inpatient procedures performed during follow-up were subsequently extracted from the National Patient Registry in Sweden and employed to identify stoma reversals, as well as any further stoma construction. Combining data from the two registries, the stoma outcome at 2 years after anterior resection was determined (any stoma *versus* no stoma). The classification method has been validated, demonstrating excellent accuracy regarding stoma outcome, as reported elsewhere^17^.

### Predictors

Within the confines of data availability, candidate predictors known before operation were selected from the SCRCR, based on previous literature reports and clinical experience. The following factors that may have contributed to a permanent stoma were included: age, sex, ASA fitness grade, BMI, minimally invasive surgery, defunctioning stoma, clinical tumour category, tumour height (distance from anal verge), neoadjuvant therapy, and hospital volume.

In the statistical models, age, BMI, tumour height, and hospital volume were managed as continuous variables. Hospital volume was calculated as the total number of anterior resections performed per year at each hospital. ASA fitness grade was treated as a categorical variable, with grades III and IV merged as there were few observations in the latter group, while retaining separation of grades I and II. Neoadjuvant treatment was divided into three groups (no treatment, preoperative radiotherapy only or preoperative chemoradiotherapy). Clinical tumour stage was appraised using the constituent parts: T category (T1–2, T3, T4, or undefined), N category (N0, N1–2, or undefined), and M category (M0, M1, or undefined). Minimally invasive surgery was treated on an intention-to-treat basis: open, or laparoscopic/robotic (including conversions).

### Statistical analysis

#### Model derivation and validation

The data were split into training (surgery during 2007–2013) and test (surgery during 2014–2015) sets. The primary outcome for the predictive models was presence of any stoma 2 years after surgery, represented as a binary variable. Internal validation was performed through 10-fold cross-validation (training set repeatedly split into training and internal validation data sets), with the incidence in each training and internal validation set kept similar.

The SuperLearner algorithm^18^ was used, which is an ensemble machine learning technique that uses cross-validation to select, in terms of predictive performance, the optimal weighted combination of a set of candidate prediction algorithms as well as to select the best single prediction method among the candidates. Thirteen predictive modelling approaches were included, ranging from traditional regression approaches such as logistic regression to modern machine learning algorithms such as random forests (*Appendix S1*). A total of 12 features were included in the models (*Table 1*). For (parametric) model selection approaches (forward/backward and least absolute shrinkage and selection operator models) all main effects, quadratic effects (of continuous variables), and all two-way interactions were included in the starting model. The interactions included, informed by clinical reasoning, were: sex by tumour height, defunctioning stoma and neoadjuvant therapy; age by tumour height, defunctioning stoma, neoadjuvant therapy, clinical tumour categories and ASA fitness grade; neoadjuvant therapy by tumour height, defunctioning stoma, clinical tumour categories, and ASA fitness grade; and defunctioning stoma by clinical tumour categories, BMI, and ASA fitness grade. For machine learning methods, all 12 co-variables were included, with no need to specify the parametric function of co-variables. The final models were assessed on test data. Area under the receiving operating characteristic curve (AUROC) with 95 per cent confidence intervals was used to evaluate overall model performance. As there is no established way of pooling SuperLearner results from multiply imputed data, complete-case data were used to fit the models.

**Table 1:** Baseline characteristics in full sample (training and test data, including missing data)

	Stoma-free	Stoma in place
	(*n* = 3991)	(*n* = 651)
Age (years)*	66.0 (52.0–80.0)	66.0 (53.0–79.0)
**Sex**		
M	2315 (58.0)	393 (60.4)
F	1676 (42.0)	258 (39.6)
BMI (kg/m^2^)*	25.5 (20.7–30.3)	25.5 (20.2–30.8)
Missing	177 (4.4)	28 (4.3)
**ASA fitness grade**		
I	1050 (26.3)	141 (21.7)
II	2320 (58.1)	373 (57.3)
III–IV	561 (14.1)	128 (19.7)
Missing	60 (1.5)	9 (1.4)
Tumour height (cm)*	10.0 (6.00–14.0)	10.0 (6.00–14.0)
Missing	28 (0.7)	3 (0.5)
**Clinical tumour category**		
cT1–T2	1178 (29.5)	127 (19.5)
cT3	2100 (52.6)	382 (58.7)
cT4	357 (8.9)	101 (15.5)
cTx	278 (7.0)	35 (5.4)
Missing	78 (2.0)	6 (0.9)
**Clinical node category**		
cN0	1811 (45.4)	243 (37.3)
cN1–N2	1757 (44.0)	350 (53.8)
cNx	397 (9.9)	55 (8.4)
Missing	26 (0.7)	3 (0.5)
**Clinical metastasis category**		
cM0	3730 (93.5)	571 (87.7)
cM1	184 (4.6)	65 (10.0)
cMx	61 (1.5)	11 (1.7)
cMissing	16 (0.4)	4 (0.6)
**Neoadjuvant therapy**		
No neoadjuvant therapy	1615 (40.5)	143 (22.0)
Radiotherapy	1716 (43.0)	345 (53.0)
Chemoradiotherapy	660 (16.5)	163 (25.0)
**Intended type of surgery**		
Open	3337 (83.6)	572 (87.9)
Minimally invasive (including conversion)	628 (15.7)	72 (11.1)
Missing	26 (0.7)	7 (1.1)
**Defunctioning stoma**		
No	921 (23.1)	42 (6.5)
Yes	3070 (76.9)	609 (93.5)
Hospital volume (anterior resections/year)*	19.4 (10.6–28.2)	19.4 (6.20–32.6)

Values in parentheses are percentages unless indicated otherwise; *values are median (i.q.r.). Data are shown for 4642 patients who had anterior resection for rectal cancer between 1 January 2007 and 31 December 2015 (and were alive at follow-up after 730 days). Presence of a stoma was determined 2 years after index surgery.

In a sensitivity analysis, patients who had died within the 2-year follow-up (still excluding 90-day mortality) were also included, with stoma status determined at the time of death. Finally, a complementary national prediction model for Swedish use only was derived, using identical methodology, but also incorporating available data on individual hospitals and healthcare region (*Appendix S1*, *Tables S1–S5* and *Figs S1–S3*).

#### Sample size

The work on sample size calculations for prediction models in binary outcomes by Riley and colleagues^19^ was applied. For this calculation, the logistic regression model with main-effects only was employed, including 19 parameters. The anticipated Cox–Snell R2 squared was set at 0.10 (indicating a conservative, high anticipated signal-to-noise ratio), the anticipated outcome proportion at 0.15 (expected permanent stoma rate), the shrinkage factor at 0.9 (recommended to decrease overfitting), and the mean absolute prediction error at 0.05 (according to convention). These assumptions rendered a minimum required sample size of 1614 observations with 243 events, with 12.74 events per candidate predictor parameter. A corresponding sample size calculation was done for the national prediction model (*Appendix S1*). All statistical analyses were performed using R version 3.6.2 (R Core Team, Vienna, Austria).

## Results

**Fig. 1:**
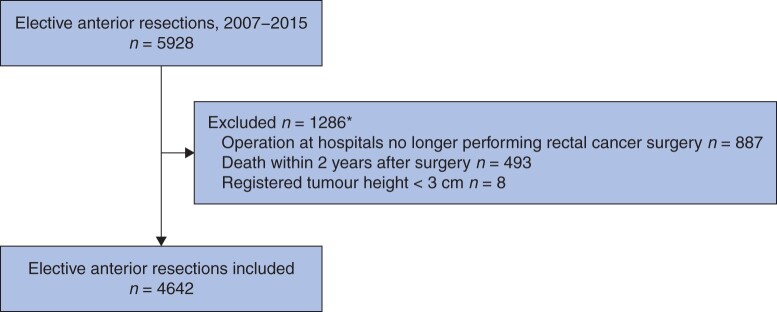
Study flow chart for patients who underwent anterior resection between 1 January 2007 and 31 December 2015, with stoma outcome determined 2 years after the index operation *Some patients fulfilled more than one exclusion criterion.

Between 1 January 2007 and 31 December 2015, a total of 4642 patients were included in the present prediction study (*Fig. 1*). Notably, 493 patients were excluded owing to death within 2 years, of whom 103 died within 90 days of surgery. After splitting the cohort, 3508 patients remained in the training data set, and the test data set comprised 1134 patients. The stoma prevalence was comparable between the two groups (13.6 and 15.2 per cent).

In contrast to patients who were free from a stoma at 2 years after surgery, those with a stoma had a higher ASA fitness grade, more advanced tumours, and more often received neoadjuvant treatment. An open surgical approach and a defunctioning stoma at the index surgery were also more common.

Excluding patients with missing values reduced the size of the training set to 3192, and that of the test set to 1087. All training set co-variables had less than 2.5 per cent missing data, except BMI with 5.3 per cent missing.

### Model development

**Fig. 2:**
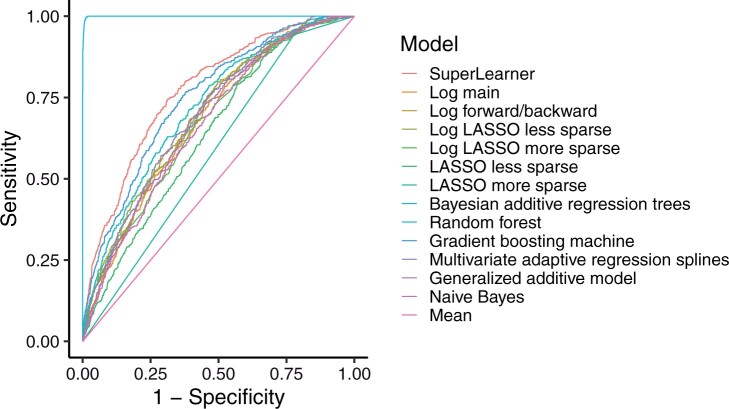
Receiver operating characteristic (ROC) curves for all models, complete-case training data (3192 patients) Log, logistic regression; LASSO, least absolute shrinkage and selection operator. Details of the prediction algorithms are available in *Appendix S1*.

The candidate prediction methods included in the SuperLearner resulted in a wide range of AUROC values when applied to the training data set (*Table 2*). The AUROC for the SuperLearner (weighted combination of all included methods) was 0.78 (95 per cent c.i. 0.75 to 0.80), whereas random forest seemed to be superior with an AUROC of 1.00 (1.00 to 1.00 ). The resulting ROC curves are shown in *Fig. 2*.

**Table 2:** Model performance based on complete-case training data (3192 patients)

	AUROC*	Predictions†
**SuperLearner**	0.78 (0.75, 0.80)	0.13 (0.08–0.17)
**Logistic regression**		
Main effects	0.68 (0.65, 0.70)	0.14 (0.09–0.18)
Forward/backward	0.70 (0.67, 0.72)	0.14 (0.07–0.18)
Refitted LASSO-selected (less sparse)	0.70 (0.67, 0.72)	0.14 (0.07–0.18)
Refitted LASSO-selected (more sparse)	0.67 (0.65, 0.70)	0.14 (0.09–0.17)
**LASSO**		
Less sparse	0.64 (0.61, 0.67)	0.10 (0.08–0.13)
More sparse	0.58 (0.57, 0.60)	0.14 (0.15–0.15)
**Bayesian additive regression trees**	0.72 (0.70, 0.74)	0.14 (0.08–0.18)
**Random forest**	1.00 (1.00, 1.00)	0.14 (0.05–0.17)
**Gradient boosting machine**	0.75 (0.73, 0.77)	0.14 (0.08–0.17)
**Multivariable adaptive regression splines**	0.70 (0.68, 0.72)	0.14 (0.07–0.18)
**Generalized additive model**	0.68 (0.66, 0.71)	0.14 (0.08–0.18)
**Naive Bayes**	0.67 (0.64, 0.69)	0.16 (0.05–0.23)
**Mean**	0.50 (0.50, 0.50)	0.14 (0.14–0.14)

Values are *mean (95 per cent c.i.) and †mean (i.q.r.). AUROC, area under receiver operating characteristic (ROC) curve; LASSO, least absolute shrinkage and selection operator. Details of the prediction algorithms are available in *Appendix S1*.

### Model validation

**Fig. 3:**
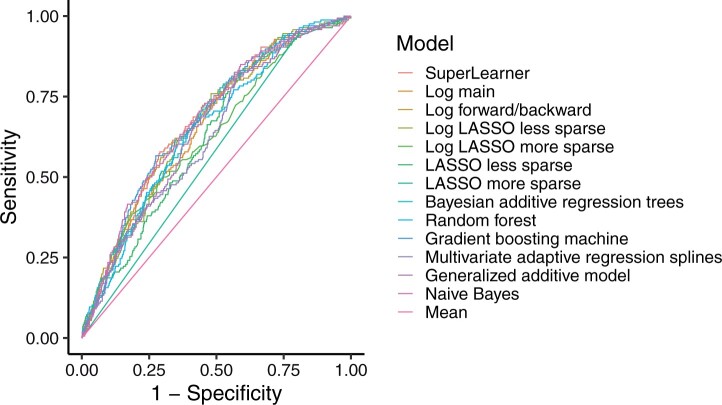
Receiver operating characteristic (ROC) curves for all models, complete-case test data (1087 patients) Log, logistic regression; LASSO, least absolute shrinkage and selection operator. Details of the prediction algorithms are available in *Appendix S1*.

**Fig. 4:**
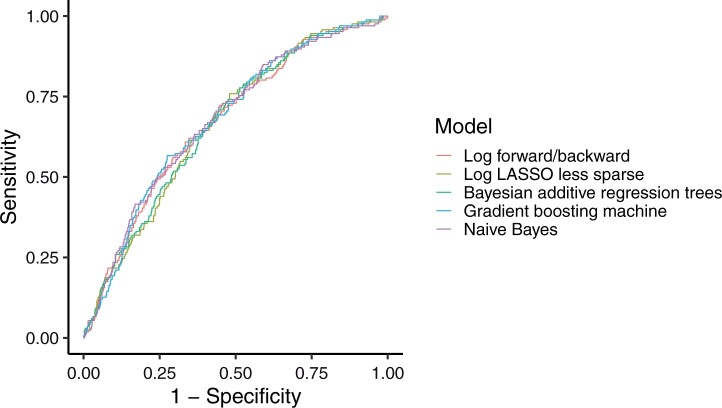
Receiver operating characteristic (ROC) curves for selected models, complete-case test data (1087 patients) Log, logistic regression; LASSO, least absolute shrinkage and selection operator. Details of the prediction algorithms are available in *Appendix S1*.

When the models fitted on the training set were applied to the test data set, a different picture with a more compressed range of values emerged, evidenced by a SuperLearner AUROC of 0.68 (95 per cent c.i. 0.63 to 0.72) (*Table 3* and *Fig. 3*). The estimated top methods turned out to be gradient boosting machine and naive Bayes, both demonstrating AUROCs of 0.68 (0.63 to 0.72). However, it is not straightforward to compute interval estimates with these methods, and very similar AUROCs of 0.67 were derived by two different types of logistic regression and Bayesian additive regression trees; a comparison of these similarly performing models is presented in *Fig. 4*. Of these models with similar results, logistic regression with a forward/backward procedure was considered the most parsimonious, with an AUROC of 0.67 (0.63 to 0.72); therefore, from here on only the results from this model will be considered. The mean stoma rate predicted using logistic regression with a forward/backward procedure was 13 (95 per cent c.i. 6 to 18) per cent. *Table 4* shows the estimated parameters. Key predictors included co-morbidity, local T category, presence of metastasis, neoadjuvant therapy, defunctioning stoma, tumour height, and hospital volume; of note, the interaction between age and metastasis was also predictive. In the sensitivity analysis including patients who died, similar results were obtained for model development and validation (*Table S6*).

**Table 3:** Model performance based on complete-case test data (1087 patients)

	AUROC*	**Predictions**†
**SuperLearner**	0.68 (0.63, 0.72)	0.13 (0.08–0.17)
**Logistic regression**		
Main effects	0.66 (0.62, 0.70)	0.13 (0.08–0.17)
Forward/backward	0.67 (0.63, 0.72)	0.13 (0.06–0.18)
Refitted LASSO-selected (less sparse)	0.67 (0.62, 0.71)	0.13 (0.07–0.17)
Refitted LASSO-selected (more sparse)	0.63 (0.59, 0.68)	0.14 (0.09–0.17)
**LASSO**		
Less sparse	0.63 (0.58, 0.67)	0.09 (0.07–0.12)
More sparse	0.57 (0.55, 0.59)	0.14 (0.15–0.15)
**Bayesian additive regression trees**	0.67 (0.62, 0.71)	0.13 (0.07–0.18)
**Random forest**	0.65 (0.61, 0.69)	0.15 (0.07–0.20)
**Gradient boosting machine**	0.68 (0.63, 0.72)	0.14 (0.08–0.18)
**Multivariable adaptive regression splines**	0.63 (0.59, 0.68)	0.14 (0.07–0.18)
**Generalized additive model**	0.65 (0.61, 0.69)	0.13 (0.08–0.17)
**Naive Bayes**	0.68 (0.63, 0.72)	0.15 (0.05–0.24)
**Mean**	0.50 (0.50, 0.50)	0.14 (0.14–0.14)

Values are *mean (95 per cent c.i.) and †mean (i.q.r.). AUROC, area under receiver operating characteristic (ROC) curve; LASSO, least absolute shrinkage and selection operator. Details of the prediction algorithms are available in *Appendix S1*.

**Table 4:** Forward/backward logistic regression model for prediction of permanent stoma after anterior resection for rectal cancer

	Odds ratio	*P*
**Sex**		
F	1.00 (reference)	
M	1.08 (0.87, 1.35)	0.496
**ASA fitness grade**		
I	1.00 (reference)	
II	0.95 (0.73, 1.24)	0.705
III–IV	1.48 (1.04, 2.09)	0.028
**Clinical T category**		
cT1–T2	1.00 (reference)	
cT3	1.34 (1.01, 1.79)	0.047
cT4	2.11 (1.39, 3.20)	<0.001
cTx	1.42 (0.89, 2.23)	0.132
**Clinical M category**		
cM0	1.00 (reference)	
cM1	2.04 (1.29, 3.13)	0.002
cMx	1.13 (0.48, 2.33)	0.753
**Neoadjuvant therapy**		
No neoadjuvant therapy	1.00 (reference)	
Radiotherapy	1.72 (1.28, 2.32)	<0.001
Chemoradiotherapy	1.36 (0.91, 2.03)	0.133
**Intended type of surgical technique**		
Open	1.00 (reference)	
Minimally invasive (including conversion)	0.67 (0.41, 1.06)	0.102
**Defunctioning stoma**		
No	1.00 (reference)	
Yes	3.34 (2.19, 5.31)	<0.001
**Age**	0.90 (0.61, 1.33)	0.585
**Tumour height**	0.81 (0.67, 0.97)	0.021
**(BMI)^2^**	1.04 (0.99, 1.09)	0.120
**Hospital volume**	0.88 (0.78, 0.99)	0.032
**(Hospital volume)^2^**	1.26 (1.15, 1.37)	<0.001
**Age: defunctioning stoma (yes)**	1.39 (0.93, 2.07)	0.106
**Age: clinical M category (cM1)**	0.57 (0.39, 0.82)	0.003
**Age: clinical M category (cMx)**	0.95 (0.44, 2.23)	0.909
**Tumour height: sex (M)**	1.20 (0.96, 1.50)	0.118

Values in parentheses are 95 per cent confidence intervals.

**Table 5:** Prediction examples for typical scenarios, using the forward/backward logistic regression model

		**Stoma risk in per cent**
**Scenario 1**		
No neoadjuvant therapy, tumour height 13 cm	Defunctioning stoma	8.4 (6.0, 11.6)
	No defunctioning stoma	2.6 (1.6, 4.3)
**Scenario 2**		
No neoadjuvant therapy, tumour height 9 cm	Defunctioning stoma	8.8 (6.4, 12.1)
	No defunctioning stoma	2.8 (1.7, 4.6)
**Scenario 3**		
Radiotherapy, tumour height 9 cm	Defunctioning stoma	14.3 (11.8, 17.2)
	No defunctioning stoma	4.7 (2.9, 7.4)

Values in parentheses are 95 per cent confidence intervals. Other categorical variables set at the mode in the complete-case training sample, and continuous variables set at the median. Other variables set at: age 66 years, male sex, BMI 25.44 kg/m2, ASA fitness grade II, cT3, cN0, cM0, hospital volume 20.1 anterior resections per year, intended open surgical technique.

### Clinical scenarios and online tool

To provide clinicians with typical predictions in a range of clinical scenarios, scenarios are provided for different rates of defunctioning stoma use, tumour height, and neoadjuvant therapy (*Table 5*). Of note, the presence of a defunctioning stoma had a substantial impact on predicted permanent stoma status, whereas tumour height and neoadjuvant therapy had a minor influence. An online calculator using the logistic regression forward/backward prediction model can be accessed at https://jennyhaggstrom.shinyapps.io/predstoma_int/, and a corresponding calculator for the national model at https://jennyhaggstrom.shinyapps.io/predstoma_nat/.

## Discussion

Prediction models for stoma prevalence at 2 years after surgery were developed and tested in this nationwide registry-based cohort of patients who had anterior resection for rectal cancer in Sweden. Several models had a similar moderate predictive ability, including the most parsimonious, which was the logistic regression model with a forward/backward procedure. The use of this model should improve the accuracy of preoperative information given to patients about stoma risk.

The main drawback of the present report is the use of registry data, including all co-variables and the predicted outcome itself. However, validation studies^15^^,^^16^ have shown that the SCRCR has excellent completeness and is highly accurate with regard to most perioperative variables, although anastomotic leakage is under-reported^20^, which may also be true for other postoperative complications^21^. The registry-based method used to determine stoma outcome (presence of a stoma at 2 years after surgery) has also been evaluated and demonstrated excellent validity^17^. Nevertheless, the SCRCR fails to capture potentially important predictors, including preoperative inflammatory status^22^^,^^23^, impaired sphincter function^24^, smoking status^25^, socioeconomic class^26^, and medication use. This potentially reduces the internal validity of the present study; still, tumour height rather than preoperative bowel dysfunction predicted the permanent stoma rate in a recent study^24^. The main advantage of the present study is the population-based design, limiting the risk of selection bias, whereas the nationwide coverage of almost a decade’s worth of surgical management reflects current practice in Sweden, resulting in high external validity and a large sample size. Moreover, the novel SuperLearner algorithm was employed; this is a cross-validation-based approach, which systematically selects the best performing prediction method among a variety of methods, including modern machine learning algorithms^18^.

There is an abundance of research on factors influencing the risk of permanent stoma after anterior resection for rectal cancer. Most studies have simultaneously analysed factors derived from before and during as well as after surgery, limiting applicability in a preoperative setting. A recent Korean study^24^, one of the few reports focusing on preoperative data, indicated that neither anal manometry nor patient-reported faecal incontinence was independently related to permanent stoma creation, whereas tumour distance from the anal verge remained the only significant predictor. However, the inclusion of patients who underwent abdominoperineal excision makes comparisons difficult. Most research highlights the importance of age^5^, co-morbidity^27^, and advanced tumour stage^2^ as risk factors known before operation, which is in line with the present findings. Prediction models for the risk of permanent stoma, irrespective of faecal diversion, are lacking. However, a Japanese study^12^ found advanced tumour stage, including metastatic disease, and the use of chemoradiotherapy to be important determinants of stoma non-reversal; these data were also used to derive a prediction model with moderate performance. Although defunctioning stomas seem to reduce symptomatic anastomotic leak rates and the need for reoperation^7^, it is recognized that such stomas intended for temporary use are frequently not reversed, with population-based studies^17^^,^^26^^,^^27^ reporting that the risk of non-reversal after anterior resection for rectal cancer is in the range 17–26 per cent. Anastomotic leakage is a major factor for permanent stoma formation^2^^,^^3^, and defunctioning stomas might reduce the risk of permanent stoma creation as a result of an anastomotic leak^4^. Only a few studies^4^^,^^13^ have attempted to quantify the impact of defunctioning stoma on the permanent stoma risk. The previous studies were limited by insufficient sample sizes to draw firm conclusions. As shown in the present study, the rate of use of defunctioning stomas for anterior resection is consistently high in Sweden; other countries have demonstrated large variation in the use of such stomas^28^, at the same time reporting no correlation with anastomotic leakage rates^8^^,^^28^. It is therefore interesting to note that the present prediction study has emphasized a defunctioning stoma as a key predictor of any stoma at 2 years after surgery.

The risk of a permanent stoma is important to patients, ranked alongside cure for the cancer itself^29^. Recent research has also indicated that quality of life is perceived as worse in patients with a permanent stoma, compared with those without^30^. An accurate prediction model of long-term stoma outcomes before surgery is therefore patient-centred and an essential part of preoperative counselling. Some patients known before operation to have high risk of permanent stoma might opt for an abdominoperineal excision instead of an anterior resection; other patients may prefer to accept the risk of an unintended permanent stoma, or can rest assured that the prediction model suggests that a stoma-free outcome is likely. It is important to note, however, that the confidence intervals are still quite wide in most instances, reflecting the inherent difficulty in predicting a permanent stoma outcome from registry-based data alone. Clinicians and patients should interpret the results cautiously, keeping in mind in particular that some predictors, unmeasured in this study, might increase the stoma risk even further, such as alcohol and smoking abuse^25^, low socioeconomic status^26^, and an inflammatory state^23^^,^^24^. Further predictive research should include other factors of potential importance that are known before operation. Long-term outcomes other than permanent stoma might have similar importance, including mortality^31^ and recurrence^32^ risks associated with anastomotic leakage after sphincter-saving surgery; moreover, similar prediction research efforts in bowel dysfunction must be considered in a patient-centred discussion^33^. An online calculator has been provided for ease of application.

## Supplementary Material

znab260_Supplementary_Data
