# Supplementary material for: Permanent stoma rates after anterior resection for rectal cancer: risk prediction scoring using preoperative variables
Source: Br J Surg. 2021 Sep 11;108(11):1388–95. doi: 10.1093/bjs/znab260 (PMC10364873; doi:10.1093/bjs/znab260)
Supplement: znab260_Supplementary_Data [file znab260_supplementary_data.zip › Supplementary information.docx]

## Supplementary information

**Candidate algorithms included in the SuperLearner**

The methods used are briefly described below. For details on Bayesian Additive Regression Trees see Tan, Y. V., & Roy, J. (2019). Bayesian additive regression trees and the General BART model. *Statistics in medicine*, 38(25), 5048-5069, and references therein; for Mixed effects regression see Pinheiro, J., & Bates, D. (2006). *Mixed-effects models in S and S-PLUS*. Springer Science & Business Media, and references therein; for the other methods see Hastie, T., Tibshirani, R., & Friedman, J. (2009). *The elements of statistical learning: data mining, inference, and prediction.* Springer Science & Business Media, and references therein.

For the SuperLearner, the function SuperLearner in the R package SuperLearner was used (Polley, E., LeDell, E., Kennedy, C. & van der Laan, M. (2019). ***SuperLearner****: Super Learner Prediction*. R package version 2.0-25.)

**Logistic regression**

*Main effects*: Standard logistic regression, including only main terms for each covariate (Log.main). Function glm in R package stats.

*Forward/Backward*: Standard logistic regression where the final model is selected by a forward-backward procedure based on the Akaike Information Criterion (Log.aic). The largest model considered included all main effects, quadratic terms for the continuous variables and all two-way interactions. Function step in R package stats.

*Refitted LASSO (less sparse)*: A Lasso logistic regression model including all main effects, quadratic terms for the continuous variables and all two-way interactions was fitted, with the tuning parameter selected by cross-validation. The terms with non-zero coefficients was selected and included in the model, which was refitted by standard logistic regression (Log.lasso.less). Function cv.glmnet in R package glmnet (Friedman, J., Hastie, T., Tibshirani, R. (2010). Regularization Paths for Generalized Linear Models via Coordinate Descent. *Journal of Statistical Software*, 33(1), 1–22.).

*Refitted LASSO (more sparse)*: Analogous to the above method but with a slightly larger tuning parameter, resulting in more shrinkage of the lasso coefficients and hence a smaller model to be refitted by standard logistic regression (Log.lasso.more).

**LASSO**

*Less sparse*: Lasso logistic regression including all main effects, quadratic terms for the continuous variables and all two-way interactions, with the tuning parameter selected by cross-validation (Lasso.less).

*More sparse*: Analogous to the above method but with a slightly larger tuning parameter, resulting in more shrinkage of the lasso coefficients (Lasso.more).

**Mixed effects regression**

*Main effects + random hospital intercept*: Mixed effect model with main effects and a random intercept for hospital (Mixed.main). Function glmer in R package lme4 (Bates, D., Maechler, M., Bolker, B., Walker, S. (2015). Fitting Linear Mixed-Effects Models Using **lme4***. Journal of Statistical Software*, 67(1), 1–48.).

*Main effects + quadratic + random hospital intercept*: Mixed effect model with main effects, quadratic effects for the continuous covariates and a random intercept for hospital (Mixed.quad).

**Bayesian Additive Regression Trees**: Bayesian tree-based machine learning method which forms an ensemble of weak prediction models to improve prediction performance (BART). An iterative Bayesian backfitting Markov chain Monte Carlo algorithm generates samples from a posterior which is used for fitting and inference. Function bartMachine in R package bartMachine (Kapelner, A., Bleich, J. (2016). **bartMachine**: Machine Learning with Bayesian Additive Regression Trees. *Journal of Statistical Software*, 70(4), 1–40.).

**Random Forest**: Tree based machine learning method which uses bagging to form an ensemble of weak prediction models to improve prediction performance (RF). Function randomForest in R package randomForest (Liaw, A. and Wiener, M. (2002). Classification and Regression by **randomForest**. *R News* 2(3), 18--22.).

**Gradient Boosting Machine**: Tree based machine learning method, which uses boosting to form an ensemble of weak prediction models to improve prediction performance (GBM). Function gbm in R package gbm (Greenwell, B., Boehmke, B., Cunningham, J. and GBM Developers (2019). **gbm**: Generalized Boosted Regression Models. R package version 2.1.5.).

**Multivariate Adaptive Regression Splines**: Adaptive regression procedure using piecewise linear splines (MARS). Function earth in R package earth (Milborrow, S. (2019). **earth**: Multivariate Adaptive Regression Splines. R package version 5.1.1.).

**Generalized Additive Model**: Additive regression where the continuous covariates are modelled as smooth functions (GAM). Function gam in R package gam (Hastie, T. (2019). **gam**: Generalized Additive Models. R package version 1.16.1.).

**Naive Bayes**: Simple probabilistic machine learning method which uses Bayes' theorem together with strong independence assumptions between the covariates (NB). Function naiveBayes in R package e1071 (Meyer, D., Dimitriadou, E., Hornik, H., Weingessel, A. and Leisch, F. (2019). **e1071**: Misc Functions of the Department of Statistics, Probability Theory Group (Formerly: E1071), TU Wien. R package version 1.7–2.).

**Mean**: Average of the outcome variable (Mean).

### Swedish model considerations

#### Predictors

The same set of predictors were used, but with the addition of healthcare region and individual hospital. Healthcare region was divided into six groups (Northern, Stockholm-Gotland, Southern, Southeastern, Uppsala-Örebro and Western), while individual hospital categories were retained in the analysis.

#### Sample size

The same principles were employed to calculate the necessary sample size as in the main model. For this calculation, the logistic regression model with main-effects only was employed, including 34 parameters. The same assumptions as in the main model rendered a minimum required sample size of 2887 observations with 434 events, with 12.74 events per candidate predictor parameter.

*Model results*

All results can be found in Supplementary Tables 1-5 and Supplementary Figures 1-3.

| **Supplementary Table 1.** Baseline characteristics in full sample (training + test data including missing data), incorporating data pertinent for Sweden only. Displaying patient demography and stoma outcome in 4642 patients who had anterior resection for rectal cancer between 1 January 2007 and 31 December 2015 (and alive at follow-up 730 days). Presence of a stoma was determined at the time point two years after index surgery | | |
| --- | --- | --- |
|  | **Stoma-free (N=3991)** | **Stoma in place (N=651)** |
| **Sex** |  |  |
| Male | 2315 (58.0%) | 393 (60.4%) |
| Female | 1676 (42.0%) | 258 (39.6%) |
| **ASA fitness grade** |  |  |
| ASA I | 1050 (26.3%) | 141 (21.7%) |
| ASA II | 2320 (58.1%) | 373 (57.3%) |
| ASA III–IV | 561 (14.1%) | 128 (19.7%) |
| Missing | 60 (1.5%) | 9 (1.4%) |
| **Clinical T stage** |  |  |
| T1–T2 | 1178 (29.5%) | 127 (19.5%) |
| T3 | 2100 (52.6%) | 382 (58.7%) |
| T4 | 357 (8.9%) | 101 (15.5%) |
| TX | 278 (7.0%) | 35 (5.4%) |
| Missing | 78 (2.0%) | 6 (0.9%) |
| **Clinical N stage** |  |  |
| N0 | 1811 (45.4%) | 243 (37.3%) |
| N1–N2 | 1757 (44.0%) | 350 (53.8%) |
| NX | 397 (9.9%) | 55 (8.4%) |
| Missing | 26 (0.7%) | 3 (0.5%) |
| **Clinical M stage** |  |  |
| M0 | 3730 (93.5%) | 571 (87.7%) |
| M1 | 184 (4.6%) | 65 (10.0%) |
| MX | 61 (1.5%) | 11 (1.7%) |
| Missing | 16 (0.4%) | 4 (0.6%) |
| **Neadjuvant therapy** |  |  |
| No neoadjuvant therapy | 1615 (40.5%) | 143 (22.0%) |
| Radiotherapy | 1716 (43.0%) | 345 (53.0%) |
| Chemoradiotherapy | 660 (16.5%) | 163 (25.0%) |
| **Healthcare region** |  |  |
| Stockholm–Gotland | 899 (22.5%) | 130 (20.0%) |
| Uppsala–Örebro | 1008 (25.3%) | 171 (26.3%) |
| Southeastern | 327 (8.2%) | 49 (7.5%) |
| Southern | 785 (19.7%) | 116 (17.8%) |
| Western | 693 (17.4%) | 115 (17.7%) |
| Northern | 279 (7.0%) | 70 (10.8%) |
| **Intended type of surgical technique** |  |  |
| Open | 3337 (83.6%) | 572 (87.9%) |
| Minimally invasive (including conversion) | 628 (15.7%) | 72 (11.1%) |
| Missing | 26 (0.7%) | 7 (1.1%) |
| **Defunctioning stoma** |  |  |
| No | 921 (23.1%) | 42 (6.5%) |
| Yes | 3070 (76.9%) | 609 (93.5%) |
| **Age (years)** |  |  |
| Median (IQR) | 66.0 (± 14.0) | 66.0 (± 13.0) |
| **Tumour height (cm)** |  |  |
| Median (IQR) | 10.0 (± 4.00) | 10.0 (± 4.00) |
| Missing | 28 (0.7%) | 3 (0.5%) |
| **Body Mass Index (kg/m^2^)** |  |  |
| Median (IQR) | 25.5 (± 4.84) | 25.5 (± 5.26) |
| Missing | 177 (4.4%) | 28 (4.3%) |
| **Hospital volume (anterior resections/year)** |  |  |
| Median (IQR) | 19.4 (± 8.80) | 19.4 (± 13.2) |
|  | | |
|  | | |

ASA = American Society of Anesthesiologists; IQR = Interquartile range

| **Supplementary Table 2.** National model performance based on complete cases training data (n=3192). | | |
| --- | --- | --- |
| **Model** | **AUROC** | **Predictions** |
|  | **Mean (95% CI)** | **Mean (IQR)** |
| **SuperLearner** | 0.80 (0.78-0.82) | 0.12 (0.07-0.17) |
| **Logistic Regression** |  |  |
| Main effects | 0.72 (0.69-0.74) | 0.14 (0.07-0.19) |
| Forward/Backward | 0.72 (0.70-0.75) | 0.14 (0.07-0.19) |
| Refitted LASSO (less sparse) | 0.73 (0.70-0.75) | 0.14 (0.06-0.19) |
| Refitted LASSO (more sparse) | 0.70 (0.68-0.73) | 0.14 (0.08-0.18) |
| **LASSO** |  |  |
| Less sparse | 0.67 (0.64-0.70) | 0.09 (0.06-0.12) |
| More sparse | 0.63 (0.61-0.66) | 0.13 (0.13-0.14) |
| **Mixed effects regression** |  |  |
| Main effects + random hospital intercept | 0.72 (0.69-0.74) | 0.14 (0.07-0.19) |
| Main effects + quadratic + random hospital intercept | 0.72 (0.69-0.74) | 0.14 (0.07-0.19) |
| **Bayesian Additive Regression Trees** | 0.73 (0.71-0.75) | 0.14 (0.07-0.19) |
| **Random Forest** | 1.00 (1.00-1.00) | 0.14 (0.05-0.17) |
| **Gradient Boosting Machine** | 0.75 (0.73-0.77) | 0.14 (0.08-0.18) |
| **Multivariate Adaptive Regression Splines** | 0.72 (0.69-0.74) | 0.14 (0.07-0.19) |
| **Generalized Additive Model** | 0.72 (0.70-0.75) | 0.14 (0.06-0.19) |
| **Naive Bayes** | 0.68 (0.66-0.71) | 0.16 (0.04-0.22) |
| **Mean** | 0.50 (0.50-0.50) | 0.14 (0.14-0.14) |

AUROC = area-under-the-receiver-operative characteristics curve; LASSO = least absolute shrinkage and selection operator; CI = confidence interval; IQR = interquartile range

| **Supplementary Table 3.** National model performance based on complete cases test data (n=1087). See Appendix for details on the prediction algorithms. | | |
| --- | --- | --- |
| **Model** | **AUROC** | **Predictions** |
|  | **Mean (95% CI)** | **Mean (IQR)** |
| **SuperLearner** | 0.67 (0.63-0.72) | 0.12 (0.07-0.16) |
| **Logistic Regression** |  |  |
| Main effects | 0.67 (0.63-0.71) | 0.13 (0.06-0.17) |
| Forward/Backward | 0.67 (0.63-0.72) | 0.13 (0.06-0.17) |
| Refitted LASSO-selected (less sparse) | 0.67 (0.63-0.72) | 0.13 (0.05-0.17) |
| Refitted LASSO-selected (more sparse) | 0.66 (0.62-0.70) | 0.14 (0.07-0.17) |
| **LASSO** |  |  |
| Less sparse | 0.65 (0.61-0.69) | 0.08 (0.06-0.10) |
| More sparse | 0.63 (0.59-0.67) | 0.13 (0.13-0.14) |
| **Mixed effects regression** |  |  |
| Main effects + random hospital intercept | 0.67 (0.62-0.71) | 0.13 (0.06-0.17) |
| Main effects + quadratic + random hospital intercept | 0.68 (0.64-0.72) | 0.13 (0.06-0.17) |
| **Bayesian Additive Regression Trees** | 0.68 (0.63-0.72) | 0.13 (0.06-0.18) |
| **Random Forest** | 0.66 (0.61-0.70) | 0.15 (0.07-0.21) |
| **Gradient Boosting Machine** | 0.68 (0.64-0.72) | 0.14 (0.08-0.18) |
| **Multivariate Adaptive Regression Splines** | 0.64 (0.60-0.69) | 0.14 (0.07-0.19) |
| **Generalized Additive Model** | 0.67 (0.62-0.71) | 0.13 (0.06-0.17) |
| **Naive Bayes** | 0.67 (0.63-0.72) | 0.16 (0.04-0.22) |
| **Mean** | 0.50 (0.50-0.50) | 0.14 (0.14-0.14) |

AUROC = area-under-the-receiver-operative characteristics curve; LASSO = least absolute shrinkage and selection operator; CI = confidence interval; IQR = interquartile range

| **Supplementary Table 4.** National Logistic regression Forward/Backward model. | | |
| --- | --- | --- |
|  | **Odds ratio (95% CI)** | **P value** |
| **Sex** |  |  |
| Female | 1.00 (Reference) |  |
| Male | 1.07 (0.86-1.34) | 0.547 |
| **ASA fitness grade** |  |  |
| ASA I | 1.00 (Reference) |  |
| ASA II | 0.93 (0.72-1.22) | 0.622 |
| ASA III–IV | 1.52 (1.07-2.15) | 0.021 |
| **Clinical T stage** |  |  |
| T1–T2 | 1.00 (Reference) |  |
| T3 | 1.45 (1.08-1.95) | 0.015 |
| T4 | 2.27 (1.48-3.48) | <0.001 |
| TX | 1.69 (1.01-2.76) | 0.040 |
| **Clinical N stage** |  |  |
| N0 | 1.00 (Reference) |  |
| N1–N2 | 0.95 (0.33-2.57) | 0.915 |
| NX | 2.23 (0.81-5.98) | 0.111 |
| **Clinical M stage** |  |  |
| M0 | 1.00 (Reference) |  |
| M1 | 2.08 (1.31-3.22) | 0.001 |
| MX | 1.10 (0.46-2.29) | 0.816 |
| **Neadjuvant therapy** |  |  |
| No neoadjuvant therapy | 1.00 (Reference) |  |
| Radiotherapy | 1.75 (1.29-2.39) | <0.001 |
| Chemoradiotherapy | 1.40 (0.92-2.13) | 0.117 |
| **Intended type of surgical technique** |  |  |
| Open | 1.00 (Reference) |  |
| Minimally invasive (including conversion) | 0.65 (0.39-1.04) | 0.084 |
| **Defunctioning stoma** |  |  |
| No | 1.00 (Reference) |  |
| Yes | 4.75 (2.53-9.94) | <0.001 |
| **Age** | 0.92 (0.63-1.36) | 0.671 |
| **Tumour height** | 0.82 (0.68-0.99) | 0.036 |
| **Hospital volume** | 0.45 (0.33-0.62) | <0.001 |
| **Hospital** |  |  |
| Ersta sjukhus | 1.00 (Reference) |  |
| Karolinska universitetssjukhuset Solna | 2.38 (1.25-4.57) | 0.009 |
| Universitetssjukhuset i Linköping | 0.63 (0.29-1.36) | 0.244 |
| Skånes universitetssjukhus Malmö | 1.29 (0.64-2.61) | 0.473 |
| Sahlgrenska universitetssjukhuset Östra | 7.16 (3.31-15.69) | <0.001 |
| Centralsjukhuset i Karlstad | 1.17 (0.61-2.23) | 0.643 |
| Universitetssjukhuset i Örebro | 0,78 (0.37-1.61) | 0.500 |
| Västmanlands sjukhus Västerås | 0.53 (0.27-1.02) | 0.056 |
| Norrlands universitetssjukhus | 0.61 (0.28-1.33) | 0.217 |
| Sunderby sjukhus | 0.49 (0.21-1.16) | 0.108 |
| Other | 0.28 (0.16-0.52) | <0.001 |
| **Age: Defunctioning stoma (**Yes**)** | 1.39 (0.93-2.05) | 0.106 |
| **Age: Clinical M stage (**M1**)** | 0.56 (0.38-0.81) | 0.002 |
| **Age: Clinical M stage (**MX**)** | 0.99 (0.44-2.35) | 0.981 |
| **Tumour height: Sex (**Male**)** | 1.21 (0.96-1.52) | 0.099 |
| **Defunctioning stoma (**Yes**): Clinical N stage (**N1–N2**)** | 0.98 (0.35-2.84) | 0.964 |
| **Defunctioning stoma (**Yes**): Clinical N stage (**NX**)** | 0.35 (0.12-1.04) | 0.053 |

ASA = American Society of Anesthesiologists

**Supplementary Table 5.** Prediction examples for typical scenarios in the Swedish setting, using the Logistic regression Forward/Backward model. Other categorical variables are set at their mode in the complete cases training sample, while continuous variables are set at their median.

| Clinical scenario |  | Stoma risk (95% CI) |
| --- | --- | --- |
| *Scenario 1* |  |  |
| No neoadjuvant therapy, tumour height 13 cm | Defunctioning stoma | 19.4% (13.1%–27.8%) |
|  | No defunctioning stoma | 4.8% (2.3%–9.7%) |
|  |  |  |
| *Scenario 2* |  |  |
| No neoadjuvant therapy, tumour height 9 cm | Defunctioning stoma | 19.6% (13.3%–28.0%) |
|  | No defunctioning stoma | 4.8% (2.3%–10.0%) |
|  |  |  |
| *Scenario 3* |  |  |
| Radiotherapy, tumour height 9 cm | Defunctioning stoma | 29.9% (21.8%–39.5%) |
|  | No defunctioning stoma | 8.1% (3.9%–16.2%) |
|  |  |  |

Other variables set at: Sex = Male, ASA fitness grade = ASA II, Clinical T stage = T3, Clinical N stage = N0, Clinical M stage = M0, Hospital = Sahlgrenska universitetssjukhuset Östra, Healthcare region = Western, Hospital volume = 43.4, Intended type of surgical technique = Open, Age = 66.

| **Supplementary Table 6.** Model performance based on complete cases test data (n = 1142) for patients who had anterior resection for rectal cancer between 1 January 2007 and 31 December 2015 (excluding deceased within 90 days of surgery, but including other deaths within followup). | | |
| --- | --- | --- |
| **Model** | **AUROC** | **Predictions** |
|  | **Mean (95% CI)** | **Mean (IQR)** |
| **SuperLearner** | 0.66 (0.62-0.70) | 0.13 (0.09-0.17) |
| **Logistic Regression** |  |  |
| Main effects | 0.66 (0.62-0.70) | 0.15 (0.09-0.20) |
| Forward/Backward | 0.65 (0.61-0.69) | 0.15 (0.08-0.21) |
| Refitted LASSO-selected (less sparse) | 0.66 (0.62-0.70) | 0.15 (0.08-0.21) |
| Refitted LASSO-selected (more sparse) | 0.61 (0.57-0.65) | 0.17 (0.16-0.16) |
| **LASSO** |  |  |
| Less sparse | 0.62 (0.58-0.66) | 0.10 (0.07-0.13) |
| More sparse | 0.59 (0.56-0.63) | 0.15 (0.17-0.17) |
| **Bayesian Additive Regression Trees** | 0.66 (0.62-0.70) | 0.15 (0.08-0.21) |
| **Random Forest** | 0.66 (0.62-0.70) | 0.17 (0.08-0.24) |
| **Gradient Boosting Machine** | 0.67 (0.63-0.71) | 0.16 (0.10-0.20) |
| **Multivariate Adaptive Regression Splines** | 0.65 (0.61-0.69) | 0.17 (0.08-0.23) |
| **Generalized Additive Model** | 0.65 (0.61-0.69) | 0.15 (0.08-0.19) |
| **Naive Bayes** | 0.66 (0.62-0.70) | 0.18 (0.05-0.28) |
| **Mean** | 0.50 (0.50-0.50) | 0.16 (0.16-0.16) |

AUROC = area-under-the-receiver-operative characteristics curve; LASSO = least absolute shrinkage and selection operator; CI = confidence interval; IQR = interquartile range
